# Mental health burden of patients with diabetes before and after the initial outbreak of COVID-19: predictors of mental health impairment

**DOI:** 10.1186/s12889-021-12101-z

**Published:** 2021-11-11

**Authors:** Sheila Moradian, Martin Teufel, Lisa Jahre, Venja Musche, Madeleine Fink, Hannah Dinse, Adam Schweda, Benjamin Weismüller, Nora Dörrie, Susanne Tan, Eva-Maria Skoda, Alexander Bäuerle

**Affiliations:** 1 Clinic for Psychosomatic Medicine and Psychotherapy, University of Duisburg-Essen, LVR University Hospital, 45147 Essen, Germany; 2 Department of Endocrinology, Diabetes and Metabolism, University of Duisburg-Essen, University Hospital Essen, 45147 Essen, Germany

**Keywords:** Mental health, COVID-19, Diabetes, Changes in mental health, Anxiety, Depression

## Abstract

**Background:**

The COVID-19 pandemic is affecting people’s mental health worldwide. Patients with diabetes are at risk for a severe course of illness when infected with SARS-CoV-2. The present study aims to retrospectively examine mental health changes in patients with diabetes in Germany before and after the initial COVID-19 outbreak, and to furthermore explore potential predictors of such changes.

**Methods:**

Over the course of eight weeks from April to June 2020, 253 individuals diagnosed with diabetes participated in an online cross-sectional study. Participants completed an anonymous survey including demographics, depression (PHQ-2) and generalized anxiety symptoms (GAD-2), distress (DT), and health status (EQ-5D-3L). In addition, all instruments used were modified to retrospectively ask participants to recall their mental health and health status before the outbreak had started. Additionally examined factors were COVID-19-related fear, trust in governmental actions to face the pandemic, and the subjective level of information about COVID-19.

**Results:**

This study shows a significant increase in prevalence of depression symptoms, generalized anxiety symptoms and distress, as well as significantly decreased health statuses in diabetes patients after the initial COVID-19 outbreak. Increased depression symptoms, generalized anxiety symptoms and distress were predicted by COVID-19-related fear, whereas trust in governmental actions to face COVID-19 predicted higher depression symptoms.

**Conclusions:**

The results indicate a negative impact of the initial COVID-19 outbreak on mental health and health status in patients with diabetes. In order to improve the efficacy of psychological support strategies for diabetes patients during the pandemic, possible predictors of mental health impairment such as the aforementioned should be examined more thoroughly and addressed more openly.

**Supplementary Information:**

The online version contains supplementary material available at 10.1186/s12889-021-12101-z.

## Background

Ever since its outbreak in China, the newly identified disease termed COVID-19 (coronavirus disease − 19) has spread rapidly through the whole world. The spread of the underlying novel virus SARS-CoV-2 (severe acute respiratory syndrome coronavirus type 2) was officially declared as a global pandemic on March 11th 2020 [1] and has impacted every nation with a few exceptions [2]. Over 131 million infections have been confirmed worldwide, entailing severe disease and almost 2.9 million deaths (until May 2021 [3]). Among confirmed SARS-CoV-2 infections, the actual number of infections is likely to be underestimated, meaning that a number of cases have probably gone undetected and the number of unreported cases is even higher [4].

To reduce the transmission of SARS-CoV-2, many countries announced several restrictions, as vaccinations and medications were still lacking in the beginning. These restrictions included, among others, the shutdown of public facilities, a partial prohibition of social gatherings, the restriction of entry and intensification of border controls, as well as the promotion of safety behaviour. The restrictions, while abrupt and unprecedented, were considered necessary to reduce infections in order to prevent capacity overload in hospitals and to protect risk groups [5].

These changes in everyday-life, alarming mortality levels in some countries and related media coverage had a large impact, not only on public life and the economic development, but also on people’s mental health [6, 7]. Recently published international literature investigating the mental health of different populations in the context of the pandemic, showed elevated prevalence in depression, anxiety and stress symptoms [8–13]. These findings are in line with recent research from Germany, which points toward increased prevalence of generalized anxiety symptoms, depression symptoms, distress and COVID-19 related fear in the German population during the first lock-down [14]. Furthermore, depression, anxiety and distress symptoms in individuals with pre-existing mental illnesses appeared to have worsened considerably [15]. Thus, pre-existing mental illness appears to be a risk factor for psychological burden, as well as being female, at younger age, a student or unemployed [16, 17].

Other risk factors for psychological burden in the context of COVID-19 include pre-existing somatic diseases like diabetes, arterial hypertension, cardiovascular or respiratory disorders [18, 19]. The pre-existing medical conditions of several somatic diseases can lead to a more severe course of COVID-19 [20] and thus they were defined as high-risk morbidities for COVID-19 [21, 22]. Diabetes is a definite risk factor for a severe course of COVID-19 [23]. This chronic metabolic disease is characterized by increased levels of blood glucose, affects about 422 million people worldwide and is directly attributed to 1.6 million deaths each year [24]. Several studies reported diabetes patients to be two to three times more likely to need intensive care, when infected with SARS-CoV-2, compared to patients with less severe disease, as well as a higher mortality in diabetes patients with COVID-19 [25, 26]. In recent literature a two-way interaction between COVID-19 and diabetes is stated, in which COVID-19 leads to worsening of dysglycemia and diabetes increased severity in COVID-19 courses [27]. Hence, patients appear to carry a double burden and need to be very careful to follow safety behaviour such as hand-washing or physical distancing [28] in order to minimize the possibility of infection and a severe course of COVID-19. A recent study showed that patients with diabetes reported higher COVID-19-related fear and more safety behaviour than matched controls [29]. Moreover, Joenson et al. [30] found that diabetes patients worried about not being able to manage their illness and treatment if infected with COVID-19.

Consequently, this increased risk for developing a severe course of COVID-19 because of a chronic somatic disease aggravates psychological burden due to COVID-19 [12, 31]. In addition to this pandemic stress, individuals with chronic diseases such as diabetes already have an increased psychological burden due to their medical condition [32]. The everyday-life of diabetes patients is characterized by challenges and complications resulting in considerable emotional distress [33], which can lead to a higher risk of mental disorders: Several studies showed higher prevalence of mental health issues in individuals with diabetes compared to individuals without diabetes [34], including depression and anxiety disorders [35–37]. Importantly, depression and anxiety symptoms may be associated with lower treatment adherence, which can lead to worse glycemic control and, ultimately, adverse diabetes outcomes [38]. As pre-existing mental health illness appears to be a risk factor for psychological burden [15], it can be assumed that patients with diabetes suffer from various stress factors during the COVID-19 pandemic.

Thus, the COVID-19 pandemic poses a particular challenge to patients with diabetes by adding uncertainty and distress to an already stressful pre-existing condition. In order to provide adequate support for patients with diabetes it is important to understand how the COVID-19 outbreak affects them and if there are risk and protective factors. In recent investigations COVID-19-related fear, pre-existing mental illness, trust in governmental actions and the subjective level of information appeared to predict mental health impairment [14, 39]. Retrospective assessments of patients were the approach chosen because, due to the sudden onset of the pandemic, no pre-pandemic data were available from this specific sample As patients with diabetes suffer from various stress factors, such as an objectively increased risk of a severe course of COVID-19 and pre-existing psychological burden, their mental health and health status might be impaired since the outbreak of COVID-19. We expect the aforementioned factors to negatively impact mental health and health status of patients with diabetes.

The increasing relevance of this issue is mirrored by the rising number of publications on the subject of mental health changes during the COVID-19 pandemic. Rather than focusing on the general health effects of COVID-19 on a population, since several representative studies have already been conducted, the aim of this study was to examine depression symptoms, generalized anxiety symptoms, distress and health status after and, retrospectively, before the outbreak of COVID-19 in individuals with diabetes. Furthermore, we aim to investigate predictors of health changes in patients with diabetes during the pandemic, in order to provide relevant insight to preventative and acute mental health services for vulnerable groups.

## Methods

### Procedure and participants

The cross-sectional study was conducted over eight weeks from April 9th to June 3rd of 2020 after the initial outbreak of COVID-19 in Germany. Data from 253 participants with diabetes were collected via an online survey. Distribution took place through diabetes-centered online channels (e.g. online newspaper) and social media channels (e.g. Facebook), as well as print media. A response rate could not be determined due to the recruitment method. The completion rate of participants who started the survey was 77.01%. Eligibility requirements for participants was a diagnosis of diabetes mellitus type 1, type 2 or other specific diabetes, age ≥ 18 years, good command of the German language, and internet access. Electronic informed consent was obtained before participants started the online survey. Study participation was anonymous, voluntary, and could be terminated at any time without any negative consequences for the participant. The study was approved by the Ethics Committees of the University Hospital Essen (20–9307-BO).

### Measures

The survey was composed of self-generated items regarding socio-demographic data, medical details and attitudes towards COVID-19, as well as validated clinical instruments and their adapted versions, assessing mental and general health variables.

#### Socio-demographic and medical details

Participants were asked to give information on gender, age, marital status, education, employment and population of their residence. Diagnosed diabetes type, quality of diabetes control, accompanying somatic illnesses and mental illness were also assessed.

#### Patient health Questionnaire-2 (PHQ-2)

The PHQ-2 consists of two items assessing depressive symptoms over the past two weeks on a four-point Likert scale (0 = “never” to 3 = “nearly every day” [40]). The cut-off for major depression symptoms is a sum score of ≥3 points. Internal consistency was high, with Cronbach’s α = .842.

#### Generalized anxiety disorder Scale-2 (GAD-2)

The GAD-2 is composed of two items screening for generalized anxiety symptoms over the past two weeks on a four-point Likert scale (0 = “never” to 3 = “nearly every day” [41, 42]). Sum scores of ≥3 indicate severe generalized anxiety symptoms. Internal consistency was high, with Cronbach’s α = .835.

#### Distress thermometer (DT)

The visual analogue scale of the Distress Thermometer was used to measure distress in the past week (0 = “no distress” to 10 = “extreme distress” [43]). The cut off for elevated distress is a score of > 4 points.

#### European quality of life 5 dimensions 3 level questionnaire (EQ-5D-3L)

The visual analogue scale EQ-5D-3L was used to assess health status (0 = “the worst health you can imagine” to 100 = “the best health you can imagine” [44]).

#### Retrospective assessment of mental health and health status

PHQ-2, GAD-2, DT and EQ-5D-3L were adapted to retrospectively assess mental health and health status before the outbreak of the COVID-19 pandemic (e.g. “Before the outbreak of COVID-19 (corona virus), how often have you been bothered by any of the following problems?”). Internal consistency for PHQ-2 (pre) and GAD-2 (pre) was high, with Cronbach’s α = .815 and Cronbach’s α = .823, respectively.

**COVID-19-related fear** was assessed by the self-generated item “I worry about COVID-19”, scaled on a seven-point Likert scale (1 = “very low” to 7 = “extremely high”).

**Subjective level of information about COVID-19** was assessed by self-generated items “I feel informed about COVID-19”, “I understand the health authorities’ advice regarding COVID-19” and “I feel informed about measures to avoid an infection with COVID-19”, scaled on a seven-point Likert scale (1 = “complete disagreement” to 7 = “complete agreement”). The scale showed acceptable internal consistency with Cronbach’s α = .788.

**Trust in government** was assessed by the self-generated items “I think all governmental measures are being taken to combat COVID-19”, “I have confidence in the governmental system in Germany” and “I think Germany is well prepared to face COVID-19”, scaled on a seven-point Likert scale (1 = “complete disagreement” to 7 = “complete agreement”). Internal consistency was high, with Cronbach’s α = .836.

### Statistical analyses

Statistical analyses were realized using the statistical program for social sciences SPSS version 26 (IBM, New York, NY, USA) and R (4.0.3). For PHQ-2 and GAD-2 sum scores were calculated, as well as mean scores for the scales trust in government and subjective level of information. Normality of the sampling distribution was assumed due to large sample sizes [45]. For sociodemographic data, measures of general and mental health, as well as COVID-19-related scales descriptive statistics were conducted. Paired *t*-tests were applied to test for differences in mental health (PHQ-2, GAD-2, DT) and health status (EQ-5D-3L) before and after the initial outbreak of COVID-19 in patients with diabetes. Bonferroni adjusted alpha levels were applied. Cohen’s *d* was used as effect size, with a *d*-value around 0.2, 0.5, and 0.8 being considered as small, medium-sized, and large effect, retrospectively. Chi-square tests were calculated to investigate the differences in prevalences for PHQ-2, GAD-2 and DT below and above clinical relevant cut-off scores before and after the initial outbreak of COVID-19. Differences in depression symptoms, generalized anxiety symptoms, distress, and health status acted as dependent variables for multivariate and univariate multiple regression models. Therefore difference values for PHQ-2, GAD-2, DT and EQ-5D-3L were calculated and included as dependent variables in the models, so that possible predictors for change could be identified. The predictors mental illness, COVID-19-related fear, trust in government and subjective level of information have been investigated as possible predictors in the general population [14] and in patients with cancer [39] before and were therefore investigated as possible predictors in our study. Homoscedasticity was given for GAD-2, DT and EQ. 5D-3L, as Breusch-Pagan tests indicated, with *p* > 0.05. For this reason Huber-White standard errors were used. The level of significance was defined as α = 0.05 (two-sided tests) if not stated otherwise.

## Results

### Sample description

The sample of 253 patients diagnosed with diabetes consists of 188 women (74.3%) and 65 men (25.7%) who were aged between 18 and 44 years (49.8%) and 45 years and older (50.2%). 169 participants reported to have type 1 (66.8%), 74 type 2 (29.2%) and 10 another specific diabetes mellitus (4.0%). An overview of the sociodemographic and medical characteristics of the sample is presented in Table 1.

**Table 1:** Sociodemographic and medical characteristics

	N	%
**Sex**		
Female	188	74.3
Male	65	25.7
**Age**		
< 45 years	126	49.8
≥ 45 years	127	50.2
**Marital status**		
Single	64	25.3
Married	117	46.2
In a relationship	55	21.8
Divorced/separated	14	5.5
Widowed	3	1.2
**Educational level**		
University education	72	28.5
Higher education entrance qualification	88	34.8
Higher secondary education	55	21.7
Lower secondary education	36	14.2
No degree	2	0.8
**Employment**		
In education	15	5.9
Full employment	102	40.3
Partial employment	53	21.3
Not employed	14	5.5
Retirement	35	13.9
Sick leave	13	5.1
Other	20	7.9
**Community size (Population)**		
100,000 residents	84	33.2
20,000 residents	72	28.5
5000 residents	41	16.2
< 5000 residents	56	22.1
**Diabetes mellitus diagnosis**		
Type 1 diabetes	169	66.8
Type 2 diabetes	74	29.2
Other specific diabetes	10	4.0
**Assessment of diabetes control**		
Good	126	49.8
Average	107	42.3
Not good	14	5.5
I can’t tell	6	2.4
**Accompanying illness (es)**		
None	122	48.2
One	54	21.3
Two	30	11.9
More than two	47	18.6
**Mental illness**		
No	184	72.7
Yes	69	27.3
**Total**	253	100

### Differences in mental health and health status before and after the initial outbreak of COVID-19

**Fig. 1:**
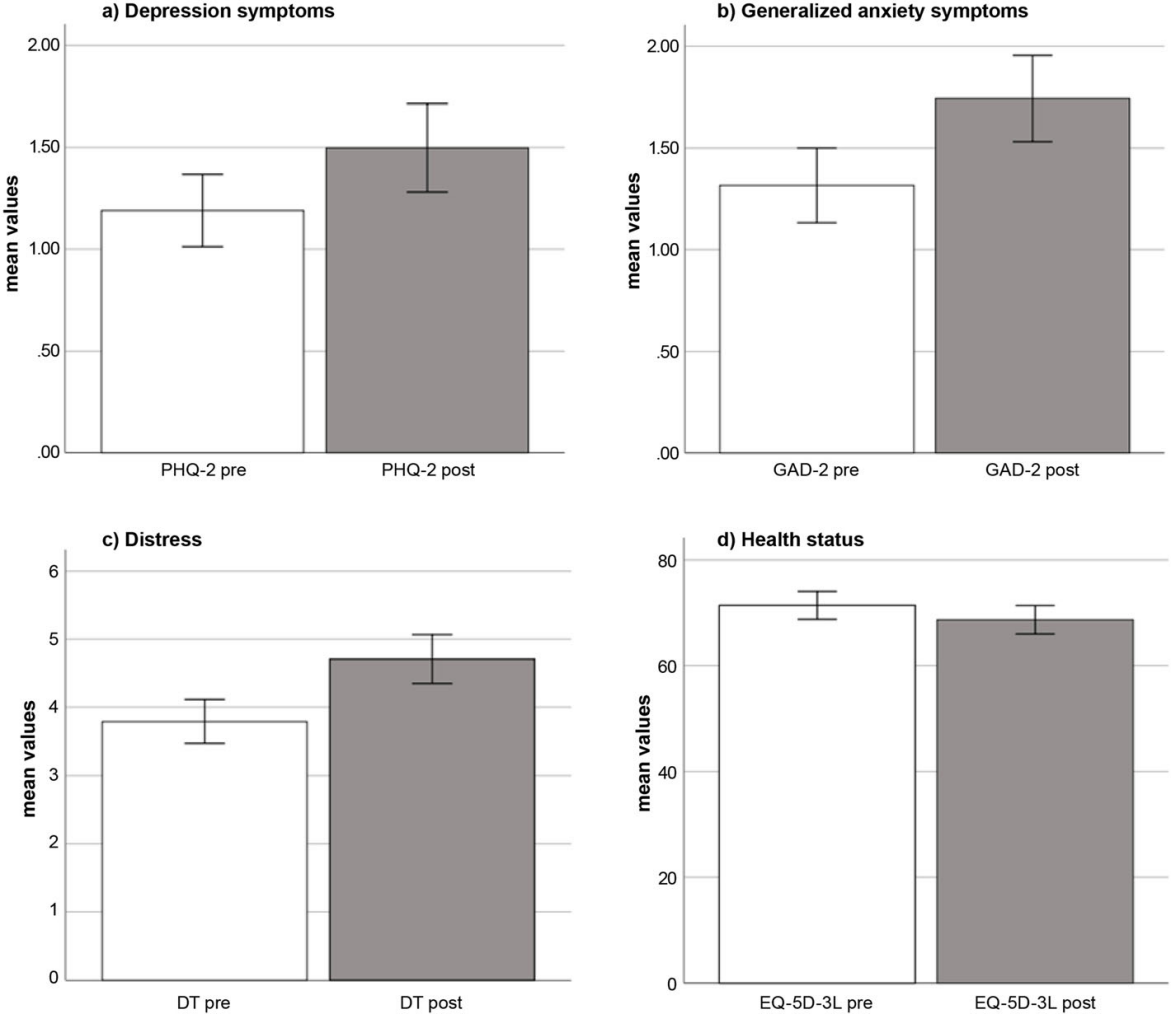
Mental health and health status mean scores before and after the initial COVID-19 outbreak. Mean values and 95% CI as error bars before (pre) and after (post) the outbreak of COVID-19 of PHQ-2: Patient Health Questionnaire-2, M_PHQ-2 pre_ = 1.19 vs. M_PHQ-2 post_ = 1.50 (a); GAD-2: Generalized Anxiety Disorder Scale-2, M_GAD-2 pre_ = 1.32 vs. M_GAD-2 post_ = 1.74 (b); DT: Distress Thermometer, M_DT pre_ = 3.79 vs. M_DT post_ = 4.71 (c); EQ-5D-3L: European Quality of Life 5 Dimensions 3 Level, M_EQ-5D-3L pre_ = 71.40 vs. M_EQ-5D-3L post_ = 68.68 (d). All differences between mean scores were significant (*p* < .005)

The results of paired *t*-tests comparing mean values of PHQ-2, GAD-2, DT, and EQ-5D-3L before and after the initial outbreak of COVID-19 revealed a significant increase in depression symptoms, *t*(252) = 5.70, *p* < .001, *d* = 0.185, generalized anxiety symptoms, *t*(252) = 4.64, *p* < .001, *d* = 0.263, distress, *t*(252) = 7.06, *p* < .001, *d* = 0.330, and a decrease in health status, *t*(252) = − 3.51, *p* = .002, *d* = 0.127. The mean scores for each scale before and after the initial outbreak of COVID-19 are presented in Fig. 1.

### Prevalence of depression symptoms, generalized anxiety symptoms, and distress before and after the initial COVID-19 outbreak

The prevalence of major depression symptoms increased from 11.9% before the COVID-19 outbreak to 21.3% after the initial outbreak (χ^2^ = 7.55, *p* = .006). For severe generalized anxiety symptoms, an increase from 13.8 to 22.9% could be shown (χ^2^ = 6.38, *p* = .012). There was an elevation in prevalence of elevated distress from 53.8% before the pandemic to 65.6% after the initial outbreak of COVID-19 (χ^2^ = 6.91, *p* = .009). For an overview, see Table 2. Results for Odd’s ratio can be found in the supplementary materials.

**Table 2:** Prevalence of depression symptoms, generalized anxiety symptoms, and distress before and after the initial COVID-19 outbreak in patients with diabetes

	Before COVID-19 outbreak *N* (%)	After COVID-19 outbreak *N* (%)	χ^2^	*p*
PHQ-2			7.55	.006
<3	223 (88.1%)	199 (78.7%)		
≥3	30 (11.9%)	54 (21.3%)		
GAD-2			6.38	.012
<3	218 (86.2%)	195 (77.1%)		
≥3	35 (13.8%)	58 (22.9%)		
DT			6.91	.009
<4	117 (46.2%)	87 (34.4%)		
≥4	136 (53.8%)	166 (65.6%)		
Total	253 (100%)	253 (100%)		

*Note:* PHQ-2 = Patient Health Questionnaire-2, sum scores of ≥3 indicate major depression symptoms; GAD-2 = Generalized Anxiety Disorder Scale-2, sum scores of ≥3 indicate severe generalized anxiety symptoms; DT = Distress Thermometer, scores of ≥4 indicate elevated distress

### Predictors of change in mental health and health status

Multivariate analyses revealed COVID-19-related fear as a global factor predicting changes in mental and general health, consisting of difference values for depression symptoms (PHQ-2), generalized anxiety symptoms (GAD-2), and distress (DT), and health status, described by EQ-5D-3L (*F*(4, 245) = 7.44, *p* < .001).

Tables 3, 4, 5 and 6 show the results of the univariate multiple regressions models predicting changes in mental and general health.

**Table 3:** Regression Coefficients Predicting an Increase in PHQ-2

	*b*a	*SE*a	*t*-value	*p*-value
Intercept	−0.250	0.580	−0.431	0.667
Mental illness	0.266	0.168	1.582	0.115
COVID-19-related fear	0.132	0.033	3.955	< 0.001
Subjective level of information	−0.176	0.092	−1.913	0.057
Trust in government	0.132	0.058	2.287	0.023

*Note*. Dependent Variable: PHQ-2 (difference between before and after the initial COVID-19 outbreak). Total *R*^*2*^ = .094, *F*(4) = 6.235, *p* < .001, *n* = 253. aUnstandardized regression coefficients. *SE*a = Huber-White standard errors

**Table 4:** Regression Coefficients Predicting an Increase in GAD-2

	*b*a	*SE*a	*t*-value	*p*-value
Intercept	− 0.303	0.524	− 0.578	0.564
Mental illness	0.068	0.179	0.380	0.704
COVID-19-related fear	0.215	0.047	4.610	< 0.001
Subjective level of information	−0.077	0.092	− 0.851	0.396
Trust in government	0.013	0.065	0.198	0.843

*Note*. Dependent Variable: GAD-2 (difference between before and after the initial COVID-19 outbreak). Total *R*^*2*^ = .103, *F*(4) = 7.566, *p* < .001, *n* = 253. aUnstandardized regression coefficients. *SE*a = Huber-White standard errors

**Table 5:** Regression Coefficients Predicting an Increase in Distress (DT)

	*b*a	*SE*a	*t*-value	*p*-value
Intercept	0.532	1.608	0.498	0.628
Mental illness	0.197	0.290	0.682	0.496
COVID-19-related fear	0.253	0.072	3.500	< 0.001
Subjective level of information	−0.115	0.148	− 0.783	0.435
Trust in government	−0.089	0.099	−0.905	0.367

*Note*. Dependent Variable: DT (difference between before and after the initial COVID-19 outbreak). Total *R*^*2*^ = .062, *F*(4) = 5.318,

**Table 6:** Regression Coefficients Predicting a Deterioration in Health Status (EQ-5D-3L)

	*b*a	*SE*a	*t*-value	*p*-value
Intercept	−9.714	6.893	−1.409	0.160
Mental illness	−3.078	2.349	−1.310	0.191
COVID-19-related fear	−0.519	0.461	−1.125	0.262
Subjective level of information	1.757	0.982	1.789	0.075
Trust in government	0.679	0.651	1.044	0.298

*Note*. Dependent Variable: EQ-5D-3L (difference between before and after the initial COVID-19 outbreak). Total *R*^*2*^ = 0.060, *F*(4) = 3.518, *p* = .008, *n* = 253. aUnstandardized regression coefficients. *SE*a = Huber-White standard errors

An increase in depression symptoms could be significantly predicted by COVID-19-related fear and trust in government, while mental illness and subjective level of information were not significant predictors. The model explains 9.4% of the variance.

COVID-19-related fear could also significantly explain an increase in generalized anxiety symptoms. Mental illness, subjective level of information and trust in government were not significant predictors, with an explained variance of 10.3%.

Increase in distress was equally significantly predicted by COVID-19-related fear, while mental illness, subjective level of information and trust in government were non-significant predictors. The explained variance of the model is 6.2%.

No significant predictors for deterioration in health status could be found.

*p* < .001, *n* = 253. aUnstandardized regression coefficients. *SE*a = Huber-White standard errors.

## Discussion

### Principal results

Individuals with diabetes have an elevated risk for developing a severe course of COVID-19 [26] and suffer from pre-existing psychological burden due to their somatic disease [32]. To understand the extent and investigate predicting factors of these individuals‘ psychological burden is essential, as they suffer from various stress factors simultaneously and may need customised psychological support during the pandemic. The present study attempted to compare mental health states of individuals diagnosed with diabetes before and after the initial COVID-19 outbreak. The results show a significant increase in the prevalence of symptoms associated with a mental health burden such as depression, generalized anxiety and distress after the initial outbreak of COVID-19 compared to symptom prevalence before. The effect sizes for all compared symptom categories were small. Further data analyses indicated two factors predicting an increase in mental health burden since the COVID-19 outbreak: High COVID-19-related fear predicted an increase in depression symptoms, generalized anxiety symptoms, and distress, whereas trust in governmental actions to face COVID-19 predicted an increase in depression symptoms. No significant predictors for the reported deterioration in diabetes patients’ health status could be found since the onset of COVID-19. Pre-existing mental illness and subjective level of information were no significant predictors of the increase in mental health burden or decrease in diabetes patients’ health status.

The findings are in line with previous research from cross-sectional studies worldwide showing elevated mental health burden during the current pandemic [8, 10, 39] and in individuals suffering from high-risk diseases [18] such as cancer [39]. Therefore, it appears that individuals with diabetes suffer strongly from psychological burden due to the pandemic. To understand the levels of stress exposure that go along with COVID-19 and a pre-existence of a somatic diseases such as diabetes is essential, as this group is already burdened with stress factors pertaining to its respective chronic somatic disease. Further stress factors due to the pandemic and the heightened risk of a severe course of COVID-19 might also increase the risk of developing mental disorders. In order to prevent such adverse effects, low-threshold access to psychological care for individuals diagnosed with diabetes seems more necessary than ever. Several approaches are already in use, such as telephone consultations or online support services for mentally burdened individuals during the ongoing pandemic [46, 47]. As these services are designed primarily for the general population, specific support for patients with diabetes is still lacking.

This study shows that increased COVID-19-related fear is a risk factor for the increase of depression symptoms, generalized anxiety symptoms and distress, which is in accordance with previous research showing similar results in the general population in Germany [14] and in cancer patients [39]. Recent literature indicates that diabetes patients report overall higher COVID-19-related fear [29]. Moreover, Kohler et al. [18] showed that the more high-risk diseases were reported, the higher the fear of COVID-19.Thus, elevated levels of COVID-19-related fear might be seen as a reflection of diabetes patients’ increased need for a sense of security. This hypothesis is supported by the fact that diabetes patients report more safety behaviour compared to individuals without diabetes [29]. In cognitive-behavioural psychotherapy it is assumed that safety behaviour arises out of a feeling of threat and can be considered as a coping strategy for anxiety [48]. Hence, COVID-19-related fear may be seen as a functional emotion which motivates a range of behaviours that reduce infection risk, but also burdens mental health to some extent. However, it is important to note here that the sample examined is not a representative sample, as the actual composition of diabetes 1 and diabetes 2 types in Germany is different from that in the sample analysed [49]. In a study with a representative sample, other effects could occur. Nevertheless, might be crucial to educate patients with diabetes about these two sides of their COVID-19-related fear in order to depathologize the emotion and, consequently, cope more adequately. However, the focus of current treatment of COVID-19 is on infection control, effective vaccine, and treatment cure rate [50, 51] and aspects of psychological impairment of high-risk groups such as patients with diabetes have yet to be thoroughly considered and addressed more openly.

Trust in government appears to predict higher depression symptoms. Further analyses indicated that trust in government and depression symptoms do not correlate, which is why the statistical significance has no topical relevance (see supplementary materials). Contrasted with previous studies with cancer patients and the general population, prior mental illness was not a predictor of an increase of psychological burden [14, 19, 39, 52]. At this point, it is important to mention that the assessment of mental illness did not differentiate between disorders, even though the spectrum of mental illnesses is broad. Besides, patients with prior mental illness might not have reported an increase in mental health burden, as it already existed before the pandemic.

### Limitations

A strong point of this study is its time of assessment, as it was conducted shortly after the first lock-down in Germany, which can be considered an early stage of the pandemic. Nevertheless, it might also be essential to assess the impact of the pandemic on mental health of diabetes patients at a different stage of the pandemic, as recent literature has shown that psychological burden and COVID-19-related fear varies between different periods of the pandemic [53, 54]. Hence, the time of study participation might impact self-reported data. As the analysed sample of the present study was too small and the survey period too short, it was not possible to assess the impacts of COVID-19 at different periods of the pandemic. Further limitations must be considered: As the data of the survey was collected via online and analogue channels, a possible selection bias cannot be ruled out. Since more participants in our sample were diagnosed with diabetes type 1, it must be questioned whether our group of participants is sufficiently representative of diabetes patients world-wide, as the majority of affected patients suffer from type 2 [55]. Also, recent literature shows that patients with type 2 diabetes had almost twice the prevalence of depression symptoms compared to patients with type 1 diabetes [56]. Further analyses of our data are in line with this observation (see supplementary materials). As the data is based on self-report, an objective verification of the diabetes diagnosis is not possible. In addition to this, more women and more patients with higher education participated in the present study, which should also be considered. The fact that non-responders could not be identified due to the anonymous approach of data assessment, adds to the aforementioned selection bias. Moreover, the results are not based on longitudinal, but on cross-sectional data. Hence, causality cannot be assumed. The instruments used to collect data regarding mental health and health status prior to the initial onset of COVID-19, were not validated as no such instruments existed at the time the study was designed and launched. To cope with this limitation, all instruments to investigate mental health and health status in the present study were adjusted to assess mental health before the initial COVID-19 outbreak retrospectively. Hence, the occurrence of recall-biased assessments should be considered. In addition, no time frame was provided for the retrospective assessment of mental health and health status before the pandemic. This clearly makes it difficult to interpret individual progressions and the different factors influencing mental health burden over the time period before and during the pandemic. Finally, it must be critically noted that determinants of change in mental health are still not clear and that reasons for mental health impairment can differ in individuals. To make generalizable observations regarding specific psychological impairments that are indeed related to COVID-19 would require an appropriate control group, such as a group of patients with diabetes in a country with very low incidence rates, and also a more representative group. Consequently, it is essential not to assume all measured effects to be caused by COVID-19. Despite of the limitations, this study provides a practicable approach to investigate mental health changes shortly after the sudden onset of the virus outbreak. Further research is recommended and should consider the abovementioned limitations while conceptualizing all instruments used.

## Conclusions

In summary, this study shows that German patients with diabetes reported increased mental health burden and impairment in health status since the COVID-19 outbreak. COVID-19-related fear appears to play an important role in diabetes patients as it was associated with increased depression symptoms, generalized anxiety symptoms, and distress. Hence, our findings could be helpful in the effort to improve the efficacy of psychological support strategies for diabetes patients suffering from the ongoing pandemic and their increased risk of a potentially severe COVID-19 course. As the crisis continues, further research is needed to assess possible causes of mental health impairment during a pandemic and potential protective factors in order to support the development of preventive treatments for individuals diagnosed with diabetes.

## Supplementary Information

**Additional file 1.**

## Data Availability

The data-sets used and analysed during the current study available from the corresponding author on reasonable request.

## Abbreviations

COVID-19: Corona Virus Disease-19
DT: distress thermometer
EQ-5D-3L: European Quality of Life 5 Dimensions 3 Level questionnaire
GAD: generalized anxiety disorder
PHQ: patient health questionnaire
SARS-CoV-2: Severe acute respiratory syndrome coronavirus type 2
