# Supplementary material for: Mental health burden of patients with diabetes before and after the initial outbreak of COVID-19: predictors of mental health impairment
Source: BMC Public Health. 2021 Nov 11;21:2068. doi: 10.1186/s12889-021-12101-z (PMC8582238; doi:10.1186/s12889-021-12101-z)
Supplement: Supplementary file 1 — Additional file 1. [file 12889_2021_12101_MOESM1_ESM.docx]

Supplementary materials

Prevalence of Depression Symptoms, Generalized Anxiety Symptoms, and Distress Before and After the COVID-19 Outbreak with Odd’s ratio

**Table 1.** Prevalence of depression symptoms, generalized anxiety symptoms, and distress before and after the COVID-19 outbreak in patients with diabetes.

|  | Before COVID-19 outbreak  *N* (%) | After COVID-19 outbreak  *N* (%) | *OR* (95% *CI*) |
| --- | --- | --- | --- |
| PHQ-2 |  |  | 2.01 (1.21, 3.40) |
| < 3 | 223 (88.1 %) | 199 (78.7 %) |  |
| ≥ 3 | 30 (11.9 %) | 54 (21.3 %) |  |
| GAD-2 |  |  | 1.85 (1.14, 3.03) |
| < 3 | 218 (86.2 %) | 195 (77.1 %) |  |
| ≥ 3 | 35 (13.8 %) | 58 (22.9 %) |  |
| DT |  |  | 1.64 (1.13, 2.39) |
| < 4 | 117 (46.2 %) | 87 (34.4 %) |  |
| ≥ 4 | 136 (53.8 %) | 166 (65.6 %) |  |
| Total | 253 (100 %) | 253 (100 %) |  |

*Note:* PHQ-2 = Patient Health Questionnaire-2, sum scores of ≥ 3 indicate major depression symptoms; GAD-2 = Generalized Anxiety Disorder Scale-2, sum scores of ≥ 3 indicate severe generalized anxiety symptoms; DT = Distress Thermometer, scores of ≥ 4 indicate elevated distress.

Prevalence of Depression Symptoms, Generalized Anxiety Symptoms, and Distress Before and After the COVID-19 Outbreak by Type of Diabetes Mellitus Diagnosis

**Table 2.** Prevalence of depression symptoms, generalized anxiety symptoms, and distress before and after the COVID-19 outbreak in patients with diabetes stratified by type of diabetes mellitus diagnosis.

|  | Before COVID-19 outbreak | | |  | After COVID-19 outbreak | | |
| --- | --- | --- | --- | --- | --- | --- | --- |
|  | Type 1 diabetes  (*N* = 169) | Type 2 diabetes  (*N* = 74) | Other specific diabetes  (*N* = 10) |  | Type 1 diabetes  (*N* = 169) | Type 2 diabetes  (*N* = 74) | Other specific diabetes  (*N* = 10) |
| PHQ-2 |  |  |  |  |  |  |  |
| < 3 | 154 (91.1%) | 61 (82.4%) | 8 (80.0%) |  | 136 (80.5%) | 55 (74.3%) | 8 (80.0%) |
| ≥ 3 | 15 (8.9%) | 13 (17.6%) | 2 (20.0%) |  | 33 (19.5%) | 19 (25.7%) | 2 (20.0%) |
| GAD-2 |  |  |  |  |  |  |  |
| < 3 | 152 (89.9%) | 58 (78.4%) | 8 (80.0%) |  | 131 (77.5%) | 56 (75.7%) | 8 (80.0%) |
| ≥ 3 | 17 (10.1%) | 16 (21.6%) | 2 (20.0%) |  | 38 (22.5%) | 18 (24.3%) | 2 (20.0%) |
| DT |  |  |  |  |  |  |  |
| < 4 | 77 (45.6%) | 36 (48.6%) | 4 (40.0%) |  | 53 (31.4%) | 29 (39.2%) | 5 (50.0%) |
| ≥ 4 | 92 (54.4%) | 38 (51.4%) | 6 (60.0%) |  | 116 (68.6%) | 45 (60.8%) | 5 (50.0%) |

*Note:* PHQ-2 = Patient Health Questionnaire-2, sum scores of ≥ 3 indicate major depression symptoms; GAD-2 = Generalized Anxiety Disorder Scale-2, sum scores of ≥ 3 indicate severe generalized anxiety symptoms; DT = Distress Thermometer, scores of ≥ 4 indicate elevated distress.

Regarding: Predictors of Change in Mental Health and Health Status

Additional analyses of the predictor trust in government in form of Pearson's product moment correlation indicated that there was no significant association between increase in depression symptoms and trust in government, *r*(251) = 0.065, *p* = 0.300.
